# Does treatment adherence correlates with health related quality of life? findings from a cross sectional study

**DOI:** 10.1186/1471-2458-12-318

**Published:** 2012-04-30

**Authors:** Fahad Saleem, Mohamed Azmi Hassali, Asrul Akmal Shafie, George A Awad, Muhammad Atif, Noman ul Haq, Hisham Aljadhey, Maryam Farooqui

**Affiliations:** 1 Department of Pharmacy, University of Baluchistan, Quetta, Pakistan/PhD candidate, Discipline of Social & Administrative Pharmacy, School of Pharmaceutical Sciences, Universiti Sains Malaysia, Minden, 11800, Penang, Malaysia; 2 Discipline of Social & Administrative Pharmacy, School of Pharmaceutical Sciences, Universiti Sains Malaysia, Minden, 11800, Penang, Malaysia; 3 Humber River Regional Hospital, 2175 Keele Street, Suite 243A, Toronto, Ontario, M6M 3Z4, Canada; 4 School of Pharmacy, Allianze University, College of Medical Sciences, Kepala Batas, 13200, Penang, Malaysia; 5 College of Pharmacy, King Saud University, Riyadh, 11451, Saudi Arabia; 6 Faculty of Pharmacy, Universiti Teknologi MARA (UiTM), Bertam, 13200, Penang, Malaysia

**Keywords:** Medication adherence, Health related quality of life, Association, Hypertension

## Abstract

**Background:**

Although medication adherence and health-related quality of life (HRQoL) are two different outcome measures, it is believed that adherence to medication leads to an improvement in overall HRQoL. The study aimed to evaluate the association between medication adherence and HRQoL.

**Methods:**

A questionnaire-based cross-sectional study design was undertaken with hypertension patients attending public hospitals in Quetta city, Pakistan. HRQoL was measured by Euroqol EQ-5D. Medication adherence was assessed by the Drug Attitude Inventory. Descriptive statistics was used to tabulate demographic and disease-related information. Spearmans correlation was used to assess the association between the study variables. All analysis was performed using SPSS 17.0.

**Results:**

Among 385 study patients, the mean age (SD) was 39.02 (6.59), with 68.8% of males dominating the entire cohort. The mean (SD) duration of hypertension was 3.010.939years. Forty percent (n=154) had a bachelors degree level of education with 34.8% (n=134) working in the private sector. A negative and weak correlation (0.77) between medication adherence and EQ-5D was reported. In addition, a negative weak correlation (0.120) was observed among medication adherence and EQ-VAS.

**Conclusions:**

Correlations among the study variables were negligible and negative. Hence, there is no apparent relationship between the variables.

## Background

The concepts of health-related quality of life (HRQoL) and medication adherence are frequently used in clinical research for assessing pharmaceutical care and treatment outcomes [1]. Adherence to drug treatment usually refers to the extent to which patients follow the instructions of their physician or other health care providers [2]. HRQoL is the assessment of physical, psychological and social dimensions of health that are influenced by an individuals experiences, beliefs, expectations and perceptions [3]. In a broader context, medication adherence is a transitional outcome variable while HRQoL is an ultimate outcome representing conclusions following a course of care [1]. This entails that a change in adherence is likely to occur first, which is subsequently followed by a change in HRQoL while assessing treatment or interventional outcomes [4]. Therefore, it can be predicted that patients who adhere to their treatment regimen should experience improvements in HRQoL and vice versa. Nevertheless, it is not wise to rule out other factors affecting medication adherence and HRQoL. For example, in the case of chronic diseases like diabetes or hypertension, adherence might be positively associated with side effects and perhaps lower HRQoL. On the contrary, for acute diseases, adherence to medication might be associated with a swift advancement in improving HRQoL. It is now known that in terms of quality of life, chronically ill individuals show lower quality of life and are less adherent to their medication [5,6].

Within this context, hypertension is a chronic disease that requires lifelong treatment. The success of therapy is thereby affected by the impact of the drug regime on the patients well-being [7,8]. Consequently, comprehensive assessment of therapy must include assessment of medication adherence and HRQoL along with the evaluation of traditional bio-physiological measures. Antihypertensive therapies should be compared for their efficacy in controlling blood pressure and also improving or at least maintaining HRQoL [9]. Regardless of the nature of studies relating HRQoL in hypertension, it is frequently reported that hypertensive patients have decreased HRQoL [10,11]. In the presence of evidence based guidelines [12,13], a challenge faced by the healthcare providers is that even in the controlled state, hypertensive patients were reported to have decreased HRQoL [14].

In the current literature, few studies have attempted to measure the association between medication adherence and HRQoL. Moreover, results from such studies provide conflicting results [1,15-17]. This may be because of the variations in assessing HRQoL and medication adherence, as well as the study population being examined [18]. In addition, more or less, such studies are often produced and reported from developed nations. Considering the unavailability of information from developing countries, the association between medication adherence and HRQoL was examined. The aim of this study was to examine the relationship between adherence and HRQoL in a hypertensive population attending public hospitals in the city of Quetta, Pakistan.

## Methods

### Study design

The study was designed as a questionnaire-based cross-sectional descriptive analysis.

### Settings

Two tertiary care hospitals [Sandamen Provisional Hospital (SPH) and Bolan Medical Complex Hospital (BMCH)] were approached for data collection. Both of the institutes are teaching hospitals and are generalised in nature, currently providing services to about 70% of the entire city.

### Ethical approval

To date, there is no ethical requirement for non-clinical observational studies in Pakistan [19]. However, permission to conduct the study was taken from the medical superintendent of the respective institutes (EA/FS/1021-2). In addition, written consent was also taken from the patients prior to data collection. The patients were informed about the research initiatives, confidentiality of their responses and their right to withdraw from the study with no penalty or effects on their treatment. In addition, the study also received its approval from the Post Graduate Research Evaluation Committee at School of Pharmaceutical Sciences, Universiti Sains Malaysia.

### Participants and sampling criteria

The National Health Survey conducted by Pakistans Medical and Research Council reported that hypertension affected 18% of the adult population in Pakistan [20]. Therefore, a prevalence-based sample of 385 hypertensive patients was selected for this study [21]. As the study was conducted in two different hospitals, 193 hypertensive patients were selected from SPH and 192 from BMCH.

Patients aged 18years and above, with confirmed diagnosis of hypertension, using antihypertensive medications for the last six months and being familiar with the national language of Pakistan (Urdu), were included in the study. Patients aged below 18 and above 80years, those with co-morbidities and mental impairments, as well as immigrants from other countries and pregnant ladies were excluded from the study. The study was conducted from July 2010 to September 2010.

### Study variables and data collection

Hospital pharmacists stationed at the cardiac unit of the respective hospitals were approached and trained by the principal researcher for data collection. Demographic information of the study respondents was taken, as written consent was provided by the patients for the initiation of the study. The Drug Attitude Inventory (DAI-10) and EuroQol quality of life scale (EQ-5D) were used to assess medication adherence and HRQoL, respectively. DAI-10 was originally available in English and was translated into Urdu (official language of Pakistan) by an independent professional translator. The translation was reconfirmed by a professor stationed at an academy of languages. However, EQ-5D was provided by the developers in Urdu. Both of the research instruments were tested for reliability (Cronbachs being 0.70 and 0.75 for DAI-10 and EQ-5D, respectively) and validity. As the originality and consistency of the two instruments were stabilised, the final versions were made available to the pharmacists. Data from the pre-test evaluation were not included in the final analysis.

### Assessment of medication adherence

DAI-10 was originally constructed by Voruganti and Awad [22] comprising ten items with responses in yes or no and scores ranging from 10 to 10. Patients with scores of 6 to 10 were reported as adherent, 0 to 5 as moderately adherent and those in the negative ranges as non-adherent [2].

### Assessment of HRQoL

The EQ-5D is a generic HRQoL instrument developed by the EuroQoL group. It consists of five dimensions that are further divided into three levels of severity. It is a standardised instrument for use as a measure of health outcome and provides a simple descriptive profile and a single index value for health status that can be used in the clinical and economic evaluation of health care as well as population health surveys [23]. The EQ-5D descriptive profile consists of five dimensions (mobility, self-care, usual activities, pain/discomfort and anxiety/depression), each of which can take one of three responses. The responses record three levels of severity (no problems/some or moderate problems/extreme problems) within a particular EQ-5D dimension. The visual analogue scale (VAS) is the other portion of EQ-5D consisting of a 20-cm health thermometer with two distinct end points, the best imaginable health state (score of 100) and the worst imaginable health state (score of 0). This information can be used as a quantitative measure of health outcome as judged by the individual respondents.

### Statistical analysis

Descriptive statistics were used to describe demographic and disease characteristics of the patients. Percentages and frequencies were used for the categorical variables, while means and standard deviations were calculated for the continuous variables. The characteristics of the whole sample, medication adherence scores and HRQoL were presented.

EQ-5D was scored using values derived from the UK general population survey reported in 1995 [24]. Medication adherence was calculated using the criteria originated by the developers [22,25]. Spearmans rank correlation test was used to measure the association between medication adherence and HRQoL. All analyses were performed using SPSS version 17.0 (SPSS Inc., Chicago, IL).

## Results

Table1 reflects the demographic characteristics and HRQoL scores among the study participants. Among the 385 study patients, the mean age (SD) was 39.02 (6.59) years, with 68.8% of males dominating the entire cohort. The mean (SD) duration of hypertension was 3.010.939years. Forty percent (n=154) had a bachelors degree level of education with 34.8% (n=134) working in the private sector. Almost 41% (n=140) had a monthly income of more than 15000 Pakistan rupees (Pk Rs) [1 Pk Rs=0.01172 $US] with 75.1% (n=289) having an urban residency.

**Table 1:** Characteristics of survey respondents and description of HRQoL scores

**Description**	**N (%)**	**Mean EQ-5D Score**	**Std Deviation**	**Mean EQ-VAS Score**	**Std Deviation**
***Age****(39.02 6.596)*					
18-27	48 (12.5)	0.5913	0.18401	66.81	5.652
28-37	186 (48.3)	0.5007	0.25706	64.68	5.862
38-47	128 (33.2)	0.4104	0.31491	59.87	7.160
>48	23 (6.0)	0.2576	0.28444	63.97	6.621
***Gende****r*					
Male	265 (68.8)	0.4677	0.28194	64.03	6.466
Female	120 (31.2)	0.4669	0.29107	63.84	6.978
***Education***					
Illiterate	9 (2.3)	0.2543	0.33554	59.44	6.521
Religious	62 (16.1)	0.3005	0.34637	60.63	6.744
Primary	7 (1.8)	0.5583	0.18048	63.57	2.992
Matric	51 (13.2)	0.4371	0.28744	64.59	7.245
Intermediate	51 (13.2)	0.5231	0.25906	65.06	5.774
BA/BSc	154 (40.0)	0.5293	0.23171	64.84	6.130
Masters	51 (13.2)	0.4835	0.28105	64.59	7.119
***Occupation***					
Jobless	97 (25.2)	0.4337	0.29882	63.24	7.077
Government official	78 (20.3)	0.4796	0.27688	64.44	7.011
Private Job	134 (34.8)	0.5295	0.23761	65.16	5.503
Businessman	76 (19.7)	0.3886	0.32602	62.36	7.080
***Income***					
Nil	97 (25.2)	0.4337	0.29882	63.24	7.077
< Pk Rs 5000	2 (0.5)	0.4210	0.33234	65.00	7.071
5000-10000	22 (5.7)	0.5628	0.19853	65.68	6.549
10000-15000	104 (27.0)	0.5231	0.23856	65.25	5.841
> 15000	160 (41.6)	0.4392	0.30643	63.34	6.735
***Locality***					
Urban	289 (75.1)	0.5113	0.25466	64.97	6.156
Rural	96 (24.9)	0.3356	0.32713	60.98	7.089
***Duration of disease****(3.010.939)*					
Less than 1year	26 (6.8)	0.5885	0.18203	67.04	4.976
1-3years	89 (23.1)	0.5158	0.25582	65.33	6.335
3-5years	124 (32.2)	0.4738	0.26777	64.35	6.106
> 5years	146 (37.9)	0.4110	0.31733	62.28	7.074
*Total Sample*	*385*	*0.4674*	*0.28444*	*63.97*	*6.621*

The mean HRQoL score was 46.7428.44 with VAS score 63.976.621 indicting poor status of life in our study respondents

The mean EQ-5D descriptive score was 0.460.28 and EQ-VAS score was 63.976.6. A total of 29 different EQ-5D health states were described by the patients (Table2). The majority of the participants (n=112, 29.1%) reported their health status (21122) indicating no problem in the second and third domain, while moderate problem in the first, fourth and fifth domain (mobility first, self-care second, usual activities third, pain/discomfort fourth and anxiety/depression being the fifth domain). There was not a single patient who stated no problem in all five domains as shown in Table2. Interestingly, poor treatment adherence (1.89) was reported in the most frequent reported heath status. Moreover, participants with best health status (11112) among the study cohort reported the worst treatment adherence (4.0).

**Table 2:** Frequency of self-reported (EQ-5D) Health States

**EQ-5D Health Status**	**N**	**% Total**
11112	1	0.3
11122	21	5.5
11123	4	1.0
11222	39	10.1
11223	8	2.1
11232	2	0.5
11233	1	0.3
12122	12	3.1
12222	6	1.6
21112	6	1.6
21121	1	0.3
21122	112	29.1
21123	12	3.1
21132	8	2.1
21212	1	0.3
21222	37	9.6
21223	13	3.4
21232	18	4.7
21233	9	2.3
22122	11	2.9
22123	5	1.3
22212	1	0.3
22222	17	4.4
22223	8	2.1
22231	1	0.3
22232	11	2.9
22233	18	4.7
22322	1	0.3
22323	1	0.3
Total	385	100

Within 29 different health states, majority (n=112, 29.1%) stated moderate difficulty in the first, fourth mad fifth domain respectively, where as they stated no difficulty in the second and third domain*.

* [(Mobility, self-care, usual activities, pain/discomfort and anxiety/depression) Domains of HRQoL in order]

### Adherence scores

The responses of patients to the DAI-10 scale are provided in Table3. DAI-10 test scores ranged between 10 and 10 with the overall mean score of 1.742.154 and median score of 2. Out of the 385 patients, 249 (64.7%) were categorised as poorly adherent and 136 (35.3%) as moderately adherent to their therapies. No patient was considered to be adhering well to their medication. Poor adherence was apparent in responses to questions 9, 5 and 2 where correct answers constituted 4.9, 15.6 and 30.4%, respectively. The correct answers were highest in response to questions 6 and 7, which were 93.0 and 76.9%, respectively.

**Table 3:** Drug Adherence Data

**Drug adherence item**	**True (%)**	**False (%)**
For me, the good things about medication outweigh the bad.	58.4	41.6
I feel uncomfortable on medication.	69.6	30.4
I take medications of my own choice.	40.3	59.7
Medications make me feel more relaxed.	60.3	39.7
Medication makes me feel tired and sluggish.	84.4	15.6
I take medication only when I am sick.	93.0	7.0
I feel more normal on medication.	76.9	23.1
It is unnatural for my mind and body to be controlled by medications.	40.0	60.0
My thoughts are clearer on medication.	4.9	95.1
By staying on medications, I can prevent getting sick.	60.0	40.0
My thoughts are clearer on medication.	4.9	95.1
By staying on medications, I can prevent getting sick.	60.0	40.0

Adherence was assessed by giving 1 to correct answer and 1 to the wrong answer. The scale measured adherence from a maximum of 10 to a minimum of 10. Any negative score was rated as non adherence, 05 as moderate adherence and 610 as adherent. Mean adherence was 1.742.15.

The Spearmans rank order correlation coefficient between total adherence and EQ-5D scores was 0.77 and total adherence and EQ-VAS scores 0.120 (Table4). Therefore, the current study findings indicate an inverse association between the included study variables. No significant difference was observed between the current study variables.

**Table 4:** Correlation coefficient (Total adherence score and EQ-VAS score)

**Spearmans Rho**	**Adherence score**	**EQ-VAS score**	**EQ-5D Score**
Correlation Coefficient	1.000	-0.120	-0.77
Sig. (2-tailed)*	-	0.169	0.132
N	385	385	385

*Correlation significant at the 0.05 level

## Discussion

The results from the present study show a weak or negligible negative correlation between medication adherence and HRQoL. The participants were also reported with decreased HRQoL and poor treatment adherence to medications. Similar results were reported in a meta analysis where hypertension patients were reported with decreased HRQoL [10]. In another study among hypertensive patients, lower medication adherence was associated with poor HRQoL in a population based survey in Brazil [14].

With the exception of the negative association, the current study findings agree with those in the literature [1,16,26,27]. However, Carbello et al. concluded that certain HRQoL domains are closely related to medication adherence in an HIV population [28]. These findings were again supported by Takemura and colleagues, who concluded that better adherence is associated with better HRQoL in their study among asthmatic patients in Japan [29].

The negative association between medication adherence and HRQoL is explainable using the theoretical model of Self-Regulation [30]. Interest and involvement of patients in improving one's own health is the key determinant of a successful medical treatment. Medication adherence is an important component of disease state management; however, it is one phase of the entire process. HRQoL, on the other hand, encircles a complex web of psychosocial characteristics that can impact a patients ability to manage their chronic disease and does not depend on a single factor. In the majority of cases, the patient observes their own behaviour and evaluates how this behaviour affects their current health status. Only if the desired results are not realised, a change in personal behaviour is initiated. If the patients are satisfied with the outcomes, they maintain status quo. A weak association from the current study is in line with the recognition that HRQoL is affected by a number of factors and is not limited to medication adherence only.

A possible explanation of this negative association can be attributed to the measurement of medication adherence and HRQoL. Although there is no gold standard for adherence and HRQoL measurement [31,32], it is always advisable to use a disease/population specific instrument. This can result in a response to small changes in medication adherence and HRQoL and perhaps can give a stronger association. Even though Cote et al. used four different instruments of HRQoL assessment (RAND-12, SF-12, HUI-2 and HUI-3) and the Morisky Medication Adherence Scale (MMAS) for assessing medication adherence, proposed a disease-specific instrument, which is in line with our suggestions [1].

The negative association that was observed here may be linked to the frequency and class of antihypertensive medication used by the patients. The efficacy of antihypertensive agents is unquestionable but certain side effects are always associated with the therapy [33,34]. Side effects in adherents can be one possible reason for the decreased HRQoL. Moreover, different antihypertensive agents affect HRQoL in a different way. Even medications from the same pharmacological class, with the same efficacy and safety profile, show different impact on HRQoL [7]. For example, a study involving two different calcium channel blockers, nifedipine and amlodipine, concluded that nifedipine had a positive effect on overall quality of life compared with no change in the amlodipine group [35].

In this context, the duration of the disease itself is very important in interpreting the association between medication adherence and HRQoL. Patients who are recently diagnosed with hypertension may experience an increased HRQoL for the first few months of therapy. However, for chronic hypertensive patients, adherence to medication might not improve HRQoL. Subsequently, HRQoL in chronic patients can be observed as maintained but this preservation is never taken as improved by the patients. This is supported by the current study results, where 146 (37.9%) and 124 (32.3%) of the respondents had hypertension for more than five years and within three to five years, respectively, and reported decreased HRQoL.

The current findings revealed that the study cohort was dominated by age group of 2837years (48.3%) and patients with bachelors level of education (40.0%). However, both treatment adherence and HRQoL are multifactor phenomenon and success or failure of therapy and overall health status are not dependent on a single factor [2,3]. Factors such as gender, low socioeconomic status, prescribed drugs, posology, lack of social support, poor patient provider relationship, cost, forgetfulness, and presence of psychological problems should also be kept in mind and evaluated before coming to a conclusion regarding treatment adherence and its effect on HRQoL.

## Conclusion

In general, the weak correlation between medication adherence and HRQoL reflects no apparent relationship. The absence of an association indicates other factors affecting HRQoL during the course of care. Further investigations of the relationship between medication adherence and HRQoL using disease-specific instruments are warranted.

### Limitations

This study has some limitations. Patients with co-morbidities were excluded from the study as the current study was conducted at a remote place with no available database. Co-morbidities, however, can modify adherence behaviour in patients as well as HRQoL. In addition, adherence was assessed by self-reported method. The use of other tools like pill counts or electronic monitoring can give sensitive results, but it is not possible to employ such methods at present as the area lacks basic infrastructure. In addition, the results were drawn from one city and cannot be generalised to the entire country.

## Competing interests

There is no conflict of interest. No funding was received for this study.

## Authors contribution

FS and NUH conducted the survey and drafted the initial manuscript. MAH, AAS and AAG designed and supervised the study. MA, HA and MF helped in statistical analysis, interpretation and manuscript revision. All authors read and approved the final manuscript.

## Pre-publication history

The pre-publication history for this paper can be accessed here:

http://www.biomedcentral.com/1471-2458/12/318/prepub
